# Synthesis and Characterization of New Multifunctional Self-Boosted Filters for UV Protection: ZnO Complex with Dihydroxyphenyl Benzimidazole Carboxylic Acid

**DOI:** 10.3390/molecules24244546

**Published:** 2019-12-12

**Authors:** Mattia Battistin, Elisa Durini, Valeria Dissette, Alessandro Bonetto, Antonio Marcomini, Elisa Casagrande, Andrea Brunetta, Paola Ziosi, Sonia Molesini, Riccardo Gavioli, Francesco Nicoli, Stefano Manfredini, Silvia Vertuani, Anna Baldisserotto

**Affiliations:** 1 Department of Life Sciences and Biotechnology, Master Course in Cosmetic Science and Technology (COSMAST), University of Ferrara, Via L. Borsari 46, 44121 Ferrara, Italy; mattiabattistin@kalis.it (M.B.); dre@unife.it (E.D.); dssvlr@unife.it (V.D.); vrs@unife.it (S.V.); bldnna@unife.it (A.B.); 2 Kalis S.r.l, Via Caodevilla 38, 31040 Onigo di Pederobba (TV), Italy; andrea.brunetta@kalis.it; 3 Department of Environmental Sciences, Informatic and Statistics, University Ca’ Foscari Venice, Vegapark, Via delle Industrie 21/8, 30175 Marghera, Venice, Italy; alessandro.bonetto@unive.it (A.B.); antonio.marcomini@unive.it (A.M.); 4 DMSN - Department of Molecular Sciences and Nanosystems, University Ca’ Foscari of Venice, Via Torino 155/b, 30172 Venice, Mestre, Italy; elisa.casagrande@unive.it; 5 Ambrosialab Srl, Via Mortara 171, 44121 Ferrara, Italy; paola.ziosi@ambrosialab.it (P.Z.); sonia.molesini@ambrosialab.it (S.M.); 6 Department of Chemical and Pharmaceutical Science, University of Ferrara, Via Fossato di Mortara 64/B, 44121 Ferrara, Italy; gvr@unife.it (R.G.); nclfnc1@unife.it (F.N.)

**Keywords:** ZnO, sunscreen, Oxisol, antioxidant, molecular combination, SPF booster, skin cancer, acne

## Abstract

The incidence of skin cancer is increasing both because of climate change and the increase in pollution than people’s incorrect habits of sun exposure. In these regards, sunscreen and photoprotection are essential tools in consenting the benefits induced by safe solar light exposition and skin cancer prevention. In this work, a new class of sunscreen filter was synthesized by chemical combination of a physical filter (ZnO) and Oxisol (dihydroxyphenyl benzimidazole carboxylic acid), an antioxidant molecule with booster effect. In this work, a new class of filters with new properties was achieved by direct functionalization of particles surface. A full characterization of this multifunctional ingredient (ZnO–Ox) was conducted: Compared with the simple mixture, the new filter acts as a multifunctional molecule showing a higher Sun Protection Factor (SPF), a better cytotoxic profile (MTT and NRU assay), and anti-acne activity. A strong reduction of photocatalytic activity of ZnO was observed, also improving the safety profile.

## 1. Introduction

Solar radiation is critical to human life and health, but in recent years, significant progress has been made to also understand the pathological effect of UV radiation from erythema, delayed tanning, photo-immunosuppression, photoaging, and photo-carcinogenesis.

Although life on earth would be impossible without the sun (which helps to give light, heat, and energy), fossil data show that all life on earth originated in the sea and that life on earth became possible only when the ozone layer covered the earth. Life became possible thanks to two different systems of nature protection: The ionosphere, which reflects X-rays into space, and the ozone layer, which absorbs the shortest UV rays. Therefore, from the beginning, life has always been linked to photoprotection [1]. 

The idea of sunscreen products was born in 1918 when Norman Paul recommended the use of ZnO as sunscreen in the Australian outback, assuming the first association between sun exposure and skin cancer in a book, published in the same year, but which was largely ignored until the early 1930s when the first sunscreen was developed industrially. Since then, the chemistry of sun filters has evolved into molecules that, if applied in the correct formulation, give an end product with excellent cosmetic properties, photo-stability, and total protection in the UVA/UVB region [2].

Several skin diseases are linked to excessive exposure to UV rays, among which photo-dermatosis is one of the most important. This disease includes a group of skin diseases exacerbated by solar radiation and can be classified into four groups: Immunologically-mediated photo-dermatosis, drug- and chemical-induced photosensitivity, photodermatosis with deficient DNA repair, and aggravated photodermatosis [1,3]. It is common practice to use sunscreen for the management of photodermatosis, and many studies have evaluated its efficacy in polymorphous light eruption (PLE), lupus erythematosus (LE), and sun urticaria (SU), with a real benefit [1].

Another skin dermatological problem related to UV radiation is photo-carcinogenesis. A 5-year study with an 8-year follow-up showed that the use of sunscreen can have a prolonged preventive effect against the development of squamous cell carcinoma and perhaps even a minimal benefit against the development of basal cell carcinoma [1,4].

Finally, UVA radiation plays an important role in photoaging and immunosuppression; in fact, even the sub erythematous dose of UV is sufficient to cause these diseases. Photoaging is triggered by receptor-initiated signaling, mitochondrial DNA mutations, protein oxidation, and telomere-based DNA damage responses. In addition, DNA photolesion, ROS (reactive oxygen species), NO (nitric oxide), uronic acid, PGE2 (prostaglandin E2), IL-10 (interleukin-10), and PAF (platelet aggregation factor) are all involved in immunosuppression [1,4].

Minerals such as zinc oxide (ZnO) and titanium dioxide (TiO_2_) are usually used as inorganic physical sunscreens in sunscreens. This class of filters is often preferred to organic filters (organic molecules with an aromatic group) due to advantages such as limited skin penetration, absence of skin irritation and sensitization, inertia of ingredients, and broad-spectrum protection [5]. Cosmetic pleasantness of product containing these filter class depends on the whitening effect which is inversely correlated to their size. Nanoparticles, and generally nano-objects, with dimensions in the nanoscale may be more biologically reactive than materials in microscale because of their higher surface area. These are the reasons why the use of nanosized ZnO and TiO_2_ particles increased recently and the safety of cosmetics containing nano-ingredients (in particular sunscreen) are particularly discussed in these years [6].

ZnO and TiO_2_ are well known as semiconductor photocatalysts extensively used in heterogeneous photocatalysis to destroy environmental pollutant. When photoactivated by UV light, these metal oxides generate high oxidizing radicals (·OH and O_2_^−^) and other reactive oxygen species (ROS), H_2_O_2_ and singlet oxygen (^1^O_2_) well known as cytotoxic and/or genotoxic agents [7].

Very recently, the practice of adding a specific additive class called “boosters” in sunscreens has been proposed in order to enhance the UV ray protection, reducing the quantity of physical and organic filter. This substance class can increase the filter’s shielding performance, with a consequent increase of the SPF [8]. In the meantime, the use of boosters may be used to decrease sun filter molecules, addressing issues related to damages to the marine environment such as coral de-coloration and so on.

From a chemical point of view, the booster effect can be obtained in a different manner; depending on the action mechanism, it is possible to distinguish between optical boosters and photochemical boosters. The first ones are based on variations in the indices of refraction of materials or in improving their dispersion; the latter are based on interactions with the energy states of classic filters [8].

Thus, there may be different kinds of boosters [9]: (1) Emollients, which have the ability to disperse the filters in a more effective and stable way; (2) polymers, such as acrylic or polyvinylpyrrolidone copolymers, which improve the thixotropic behavior of the emulsion, giving it better distribution on the skin and maintaining a thin and homogeneous layer on the skin; (3) coating of filtering substances to increase the number of light passages through films with different refractive indexes (multilayer filter effect), bead that increase the particle diameter of the nano-filters contained inside, also reducing their photo-reactivity; (4) hollow spheres that are empty spherical particles with a different refractive index than the polymeric shell and act as a UV scattering center; and finally (5) quencher, that are non-filtering substances that break down the various excited states of the filters, which are essential for stabilizing the filtering system over time.

**Figure 1:**
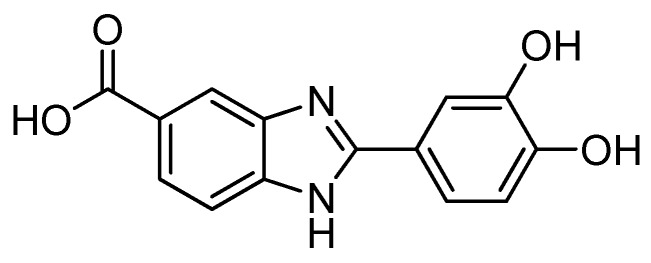
Dihydroxyphenyl benzimidazole carboxylic acid (Oxisol).

Dihydroxyphenyl benzimidazole carboxylic acid (Oxisol, University of Ferrara European Patent EP2800741) (Figure 1) is the representative of an interesting new class of UV boosters provided with a powerful radical scavenger profile [9]. Oxisol is devoid of any significant UV-filtering activity, but has an unattended UV-filtering booster capability on well-known UVB and UVA filters (both organic and inorganic) [9]. 

**Figure 2:**
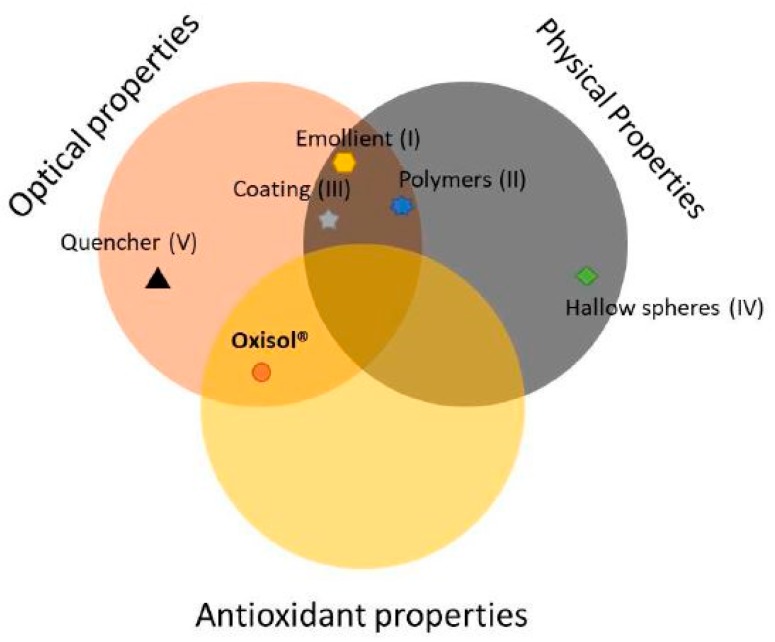
Schematic description of Oxisol location if compared with another booster. It results the only molecule with antioxidant activity.

Figure 2 efficiently describes the particularity of the Oxisol molecule. If compared with other booster classes, it results the only molecule with both antioxidant activity and optical properties.

However, during our studies, the association of Oxisol and physical UV filters (p-UVf) (TiO_2_ and ZnO) showed some limitations, including a pH range of application that can influence antioxidant activity and color change. As already reported by Nakayama [10], the functionalization of mineral oxide surface leads to an increased suspension stability, and can also act on the photocatalytic activity by improving the light adsorption mechanisms. Furthermore, zinc anti-acne properties have been well known for a century, but zinc is experiencing a second youth during recent years because of the great attention from people for natural products. It is so considered an emerging alternative acne treatment to reduce possible adverse effects of antibiotics, and in view of *Cutibacterium acnes* (*C. acnes*) strains developing resistance to conventional antibiotics. The exact mechanism is not clear, but a combination of different effects is supposed. In fact, zinc is considered to act directly on microbial inflammatory equilibrium and facilitate antibiotic absorption when used in combination. Furthermore, topical zinc alone, as well as in combination with other agents, could be effective because of its anti-inflammatory activity and ability to reduce *C. acnes* counts by inhibition of *C. acnes* lipases and free fatty acid levels. Finally, another proposed mechanism is directly linked to a suppression of sebum production by its antiandrogenic activity [11].

**Figure 3:**
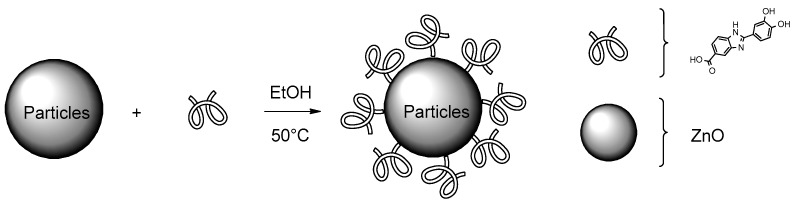
Adduct preparation (ZnO–Ox).

Taking all of this into account, in the present work, we investigated the performance on UV protection and radical scavenging activity of a molecular combination between ZnO and Oxisol (Figure 3) in order to explore possible advantages of covalently bonded booster and filter over the simple mixture. To this aim, a complete characterization was carried out also exploring the biological properties of the obtained adduct. Furthermore, to compare the unattended cytotoxicity observed, a Zn^2+^ complex was also prepared and tested in order to explore possible influences of the biological behavior of this cation.

## 2. Results and Discussion

### 2.1. Adduct Characterization (FT-IR)

**Figure 4:**
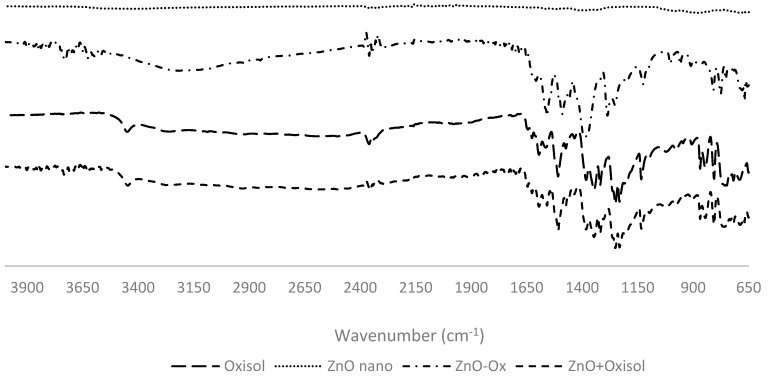
Comparison between FT-IR spectra of ZnO (ZnO nano), ZnO/Oxisol mixture (ZnO nano+Oxisol), functionalized (ZnO nano–Ox), and Oxisol (Oxisol).

The qualitative investigation of the obtained products was performed by IR spectroscopy, making a comparison between pure nanometric ZnO, pure Oxisol, nanometric ZnO and Oxisol mixture, and functionalized Oxisol (Figure 4).

**Figure 5:**
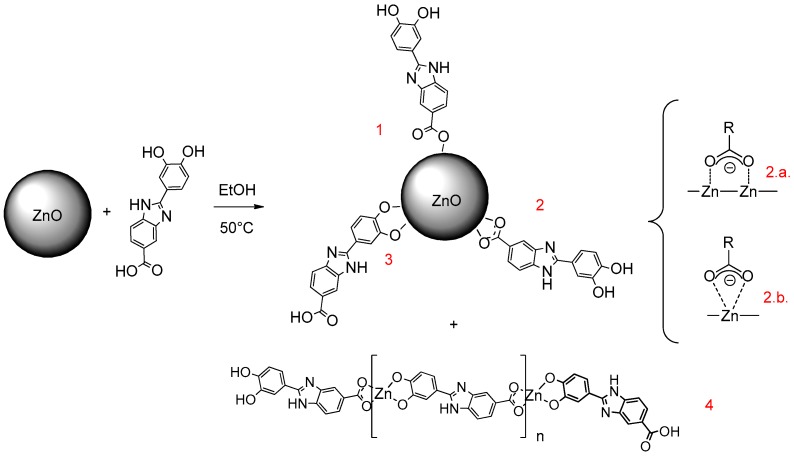
Possible addition ways of Oxisol to ZnO. Addition via carboxylic group would seem the most favored, both via bridging (**2.a.**) and chelating (**2.b.**) mechanisms. However, mechanisms through path 1 (carboxylic hydroxyl) and 2 (catechol) can occur, though less frequently. Chelation of Zn^2+^ ion can also occur also (**4**), and stoichiometric study suggests a polymeric structure of complex.

The clear difference between the spectra of Oxisol and the mixture with ZnO nano, as compared to the adduct (ZnO nano–Ox) spectra, represent a first confirmation of the addition of zinc oxide (Figure 4). In detail, it can be noted the disappearance of the peak related to ν (C=O) stretching at 1700 cm^-1^ (evident in mixture spectrum, ZnO nano+Oxisol) referred to the pure carboxylic acid. Peak splitting in anti-symmetric ν_as_ (COO^−^) stretching and ν_s_ (COO^−^) symmetric at minor wave frequencies (1400–1500 cm^−1^) is characteristic for carboxylic group coordinated with metal [12]. These changes can be assumed as diagnostic of the bond formation between Zinc and Oxisol (Figure 5). Quantitative adsorption was followed monitoring the disappearance of Oxisol in supernatant by UV-Vis spectroscopy.

### 2.2. Thermogravimetric Analysis (TGA)

**Figure 6:**
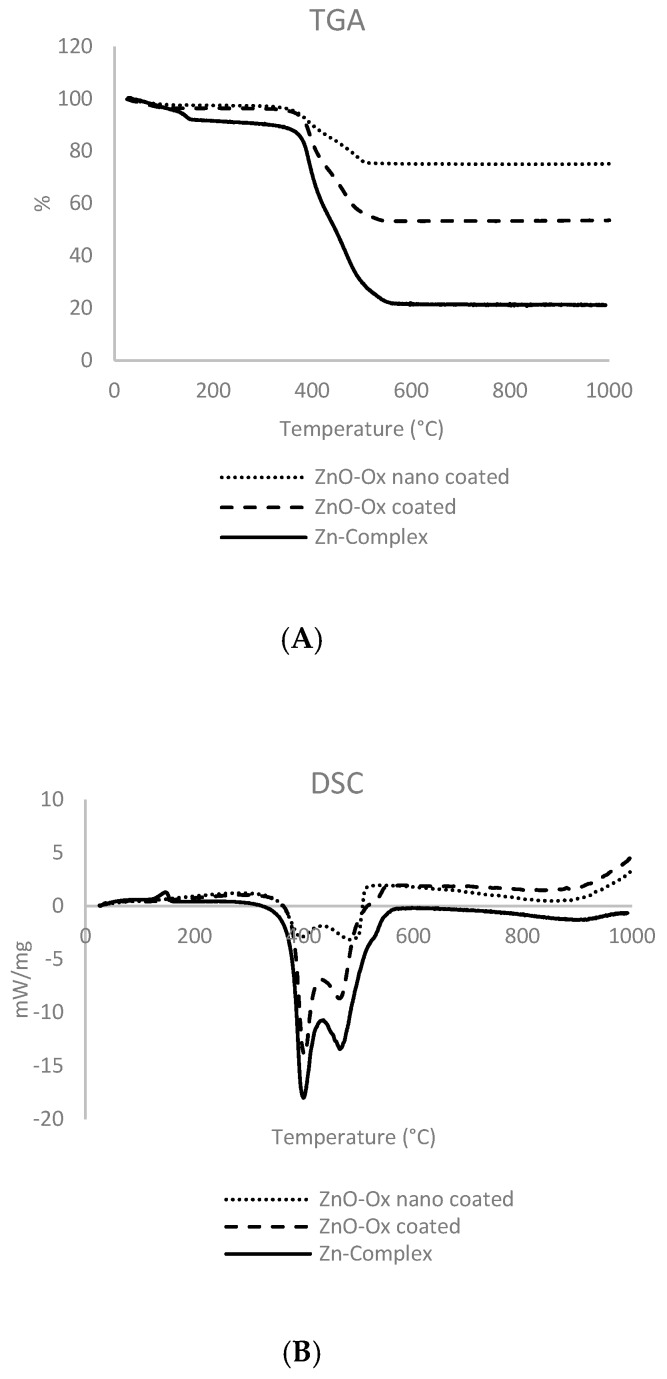
TGA (**A**) and DSC (**B**) analysis for nanometric-coated particles, non-nanometric-coated particles ZnO (ZnOOx) and complex. The two different peaks of DSC might be associated with a different kind of bond (functionalization and complexation).

Indirect and direct analysis was carried out in order to understand the stoichiometry of adduct. Below are the results obtained by TGA (Table 1) for the different adducts and zinc complex. TGA analysis are show in Figure 6A.

The obtained data show a high presence of organic fraction in adduct species. The great similarity of differential scanning calorimetry (DSC) analysis (Figure 6B) between non-nanometric adduct and complex leads to believe in a complex formation; this can explain the high organic fraction too in this material. Furthermore, these analyses give an important idea about the stoichiometry of Zn complex that would seem 1/1 Zn/Oxisol. Usually, Zn^2+^ gives tetrahedral complexes [13], which suggests that the new material (complex) presents a polymeric structure given by the coexistence of two different group (chatecol and acid) in the same molecule (Oxisol) that might lead to a double coordination with Zn^2+^.

Other confirmation of a large amount of organic fraction came from sample digestion and ICP–MS analysis. This assay confirms the organic fraction quantity, giving comparable data with TGA (Table 2).

The high presence of organic fraction was therefore confirmed. Not only the general large amount of Oxisol, but also the higher presence of this on non-nanometric ZnO instead of nanometric ZnO needed further investigation; therefore a SEM analysis of non-nanometric samples was carried out.

**Figure 7:**
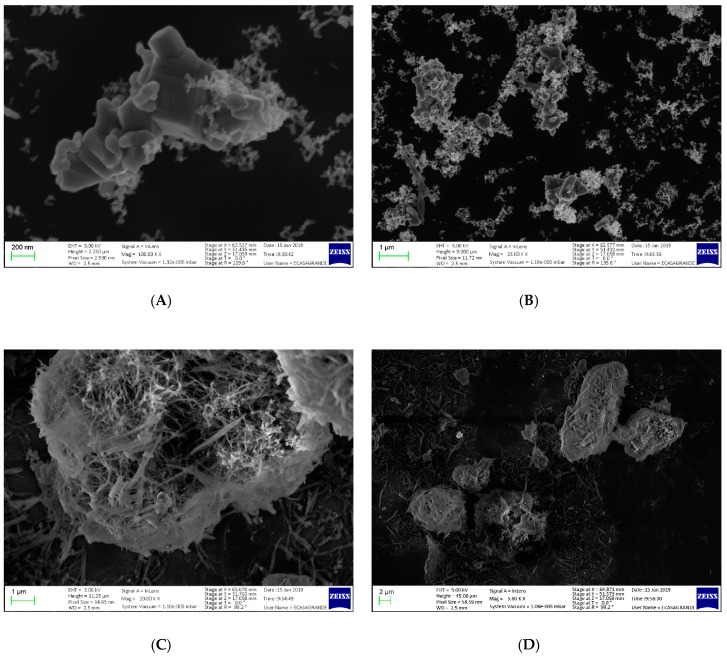
SEM image of non-nanometric ZnO (**A**,**B**) and of functionalized non-nanometric ZnO (pael **C**,**D**).

**Figure 8:**
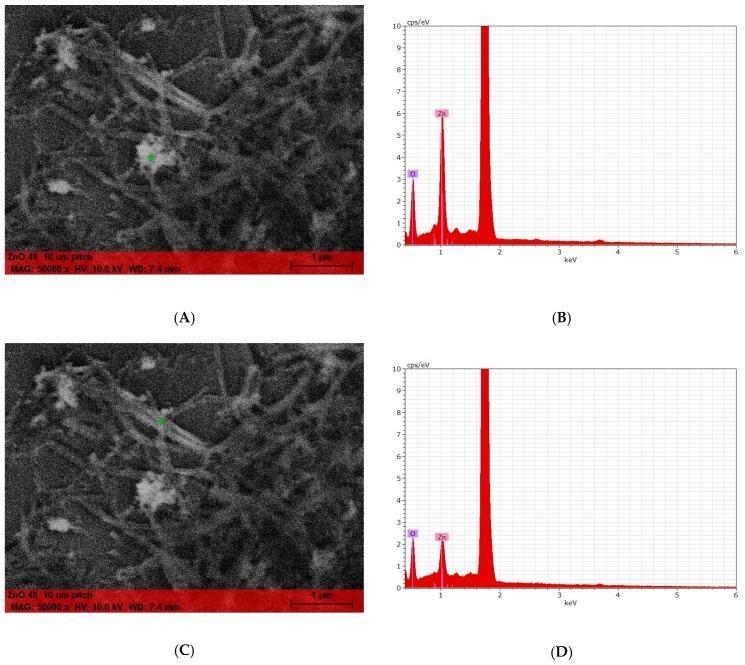
The green cross in (**A**) shows the point where the EDX analysis (**B**) was carried out (non-needle shaped particle). (**D**) The same analysis carried out for needle-shaped particle (as indicated by green cross in (**C**)).

Figure 7 show the uncoated non-nanometric ZnO (A,B) and the coated ZnO (C,D). In panels A and B, the characteristic structure of the ZnO can be observed, in addition to a certain heterogeneity of the particle size. Panels C and D, on the other hand, show a dual nature of the material: Agglomerated structures are alternated with other needle-like ones. This evidence leads us to hypothesizeze a double nature of the material obtained. In order to confirm this, a SEM–EDX analysis was performed by comparing the different Zn/O ratio between “bulk” and needle-like structures (Figure 8).

Panels A and C (Figure 8) give the image of samples analyzed. The green cross indicates the precise point analyzed (A, bulk; C, needle). Panels B and D give the corresponding analysis, where zinc and oxygen abundance are highlighted. The difference of spectra gives us a confirmation of the different nature of products, where the needle presents a greater amount of organic ligand.

**Figure 9:**
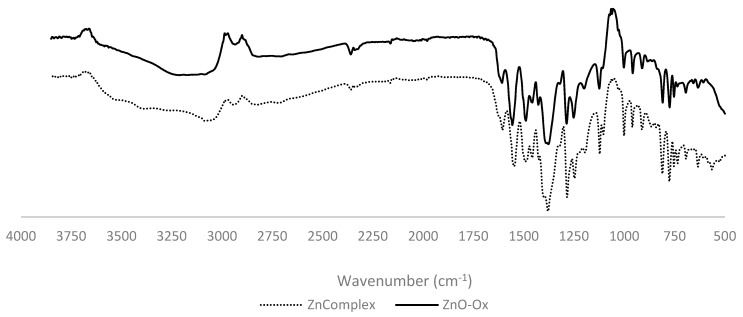
Comparison between complex and adduct spectra. Results show a similarity of spectra (95% in the region between 1700 and 650 cm^−1^); the only difference is represented by the characteristic signal of ZnO near 500cm^−1^ that confirms the co-existence of coating and complexation in the adduct.

Further confirmation of a structure different from a simple functionalization was given by ATR–FT-IR analysis. In fact, an analysis of the complex obtained by reaction between ZnCl_2_ and Oxisol was performed, confirming the high similarity (95%) between functionalized ZnO and the Zn complex (Figure 9). However, the presence of characteristic peak of ZnO at 500 cm^−1^ confirms the presence of both coating and complexation.

**Figure 10:**
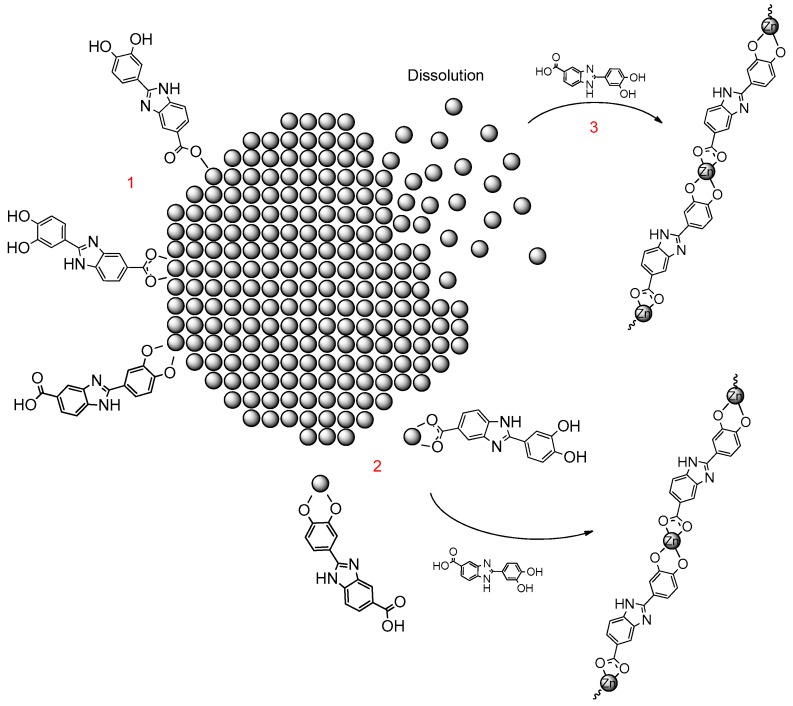
Hypothesis about zinc complexation: Ligand-promoted dissolution by Oxisol (**2**) and equilibrium shift by ion complexation, otherwise dissolution equilibrium, that naturally occurs in ZnO particles, was shifted by Oxisol complexation (**3**). Both these processes happen simultaneously with the ZnO coating (**1**).

This gives further evidence to the complex formation besides the simple coating. The mechanism could be based on a solubilization and recrystallization of ZnO due to reaction with Oxisol. This phenomena (that could efficiently explain the TGA values) can occur through two different mechanism: Ligand-promoted solution or equilibrium shift by ion complexation (Figure 10) [14].

### 2.3. Colloidal Characterization (CSA, DLS–ELS, ζ)

Despite that the new products are not a simple coating, but a mixture of coated particles and complex, Z potential and sedimentation rate were performed anyway. Results are shown in Table 3.

DLS analysis is generally in accordance with CSA analysis. Initially, a single DLS analysis was performed for pure non-nanometric ZnO. This analysis showed a high standard deviation, characteristic of heterogenous systems containing a large particle size distribution. A first indication was already observed with SEM (Figure 8A,B); therefore, to have definitive confirmation, a second analysis was carried out, analyzing the particle size at three different times. This led to obtaining a particle distribution ranging from 10 to 1600 nm (Table 4, column 1). The large population of particles in the non-nanometric ZnO sample could be the reason of its reactivity (even higher than the nanometric ZnO) with Oxisol.

Concerning sedimentation speed, the data obtained are aligned with the dimensional considerations. For pure oxides, in particular, by increasing the diameter of the ZnO particles, from the nanometric to the micrometric form, an increase in sedimentation speed is observed (from 33 to 94 μm/s, respectively). The CSA analysis shows that the modification of the morphological structure of the micronized ZnO particles after the reaction with Oxisol leads to the formation of needle-like structures, with a significant effect also on the sedimentation rate, aligning the nanometric and non-nanometric ZnO–Ox values.

**Figure 11:**
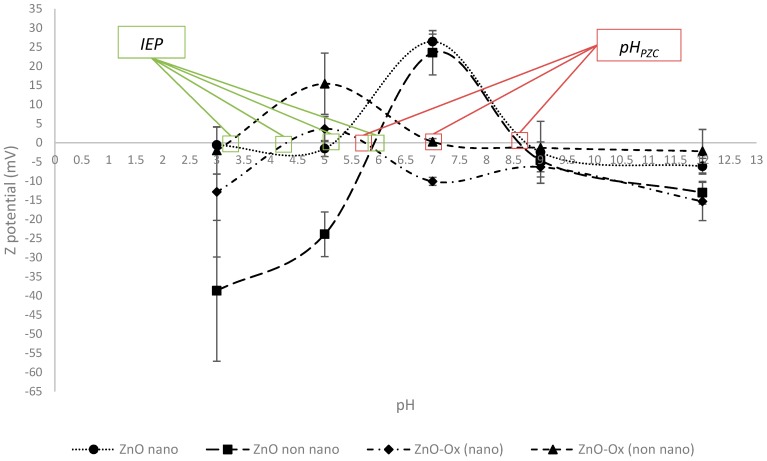
Z potential behavior for each kind of ZnO, coated and uncoated. Isoelectric point (IEP) and pH in which point zero charge is reached was reported (pH_PZC_).

Notwithstanding that the experimental evidence suggests that a new compound was achieved instead of a coating, a Z potential analysis was conducted. The data obtained are reported in Figure 11. 

The behavior of pure ZnO (nanometric and non-nanometric) follows the expected trend; in fact, the reason why the Z potential decreased for pH values under 7 could be attributable to dissolution of ZnO [15,16]. As described by Fetehah et al., the dissolution of ZnO is caused by the formation of Zn^2+^*_(aq)_* and H_2_O.

For the particles modified with Oxisol, the interpretation of the Z potential results to be a difficult task, because of the changing in the physical–chemical properties of the materials that may falsify the measure.

### 2.4. Efficacy Tests 

#### 2.4.1. SPF

Following irradiation with a solar simulator, it was observed that all the formulations tested turned out to be photostable, as the post-irradiation SPF values did not show significant changes (Table 4). The adduct (ZnO–Ox) shows a consistent SPF value in comparison with the simple mixture (ZnO+Oxisol). In fact, covalent bonding increases the SPF value by 107% for nanometric ZnO (from 2.01 to 4.17) and by 60% for non-nanometric species (from 2.08 to 3.32). Also, UVA-Protection Factor (UVA-PF) increased, but only with nanometric metal oxide was the variability significative. UVA/UVB ratio and lambda critical value did not show any significative changing, maintaining the same values for each formulation. The reason of SPF increasing could be explained by the increasing of absorption spectra of complex, like in Dye Sensitized Solar Cells (DSSC). Furthermore, the coordination of ZnO could lead to a new raw material with new optical properties, not comparable with the simple mixture.

#### 2.4.2. Photochemiluminescence (PCL)

PCL analysis was performed in order to evaluate the antioxidant activity, which is related to the amount of free phenol hydroxyl groups, and thus to the stability of the ZnO–Ox complex. These results are shown in the below Table 5.

The results of the PCL analysis show that functionalization negatively influences the antioxidant activity; in fact, it was observed that the antioxidant power is not minimally preserved. These data therefore lead to argue that the reason lies in the complex formation that inactivates Oxisol.

### 2.5. Release Test

For the functionalized particles obtained as at Section 3.2, a stability test was carried out monitoring the Oxisol released during 4.0 ± 0.5 h in the following conditions: Ethanol, ethanol and water mixture (2:3), and water at pH 2.7, 6.1, 12—conditions chosen in order to predict the behavior in the emulsion on the skin. A study on Oxisol release from the new adduct was carried out (Table 6). 

No significant release was observed in ethanol and ethanol/water environment. The most interesting results were obtained for pure water, where the maximum release was achieved for high pH, as expected. In fact, high pH values catalyze the ligand hydrolysis and, at the same time, its salification and, consequently, its solubilization in water.

The results of the Oxisol release study from the formulations containing ZnO–Ox are shown in Table 7, and consent to understand the behavior of the materials when applied in emulsion and, thus, to the skin.

### 2.6. Safety Tests 

#### 2.6.1. Photocatalytic Activity

Since the development of ROS driven by a physical filter is mainly due to a photocatalytic activity, it was investigated, by Acid Blue 9 test, if the coating of the filters under examination led to a reduction of the photocatalytic activity [17,18,19]. The results obtained from photocatalysis tests are shown in Table 8; dye absorbance was monitored after irradiation.

Data presented above show little photo-sensitization of dye. It is evident that the Oxisol coordination efficiently quenches the photocatalytic activity. The same experiment carried out in dark condition confirmed that the contribution of dye adsorption in the particle surface is very low. This phenomenon could be explained through two different mechanisms: (1) Steric hindrance generated by the ligand, and (2) the ligand injects the electron directly into the ZnO conduction band, preventing the formation of the h^+^ species in the valence band, with consequent decrease of the oxidizing power. However, the result is a strong decrease in the photo-oxidative activity of the functionalized physical filter. 

#### 2.6.2. Cytotoxicity

The cytotoxicity profile was evaluated by testing increasing concentrations of the different compounds in 3T3 cell cultures. As can it be seen in Table 9, both ZnO displayed a similar dose-dependent cytotoxic profile, with an almost complete inhibition of cell proliferation at the concentration of 100 μg/mL, while no effects were observed at the dose of 1 μg/mL.

Notably, while non-nanometric ZnO and nanometric ZnO displayed cell toxicity greater than 50% at the dose of 10 μg/mL, their functionalized counterparts did not show any toxic effects at the same concertation, suggesting that this Oxisol addition reduces their cytotoxic potential.

It can also be observed that the difference between ZnO–Ox and ZnO (in both of its forms) is statistically significant: In particular, a significant decrease (0.001< *p*-value < 0.01) was observed for the formulations at a concentration of 10 μg/mL.

The reasons on this cytotoxicity inhibition cannot be attributed only on ZnO decreasing in ZnO–Ox species, as it is not proportional with ZnO concentration (see Table 1 and Table 2). This aspect will be subjected to further investigation.

Results obtained in terms of cytotoxicity of ZnO and ZnO–Ox complexes are not surprising, since, in the literature, it is reported that zinc oxide nanoparticles have cytotoxic effects caused by their induced oxidative stress (in human dermal cells A431) [20]. But, further studies have also shown the existence of other mechanisms of action that occurs in zinc oxide cytotoxicity, i.e., the release of Zn^2+^ initiates a cytotoxic pathway that includes intracellular calcium flow, mitochondrial depolarization, and cell membrane destruction [21].

### 2.7. Solubility Test

In order to understand better the reason why the cytotoxicity considerably changed from ZnO and ZnO–Ox, a solubility test was carried out. In fact, as mentioned in the section above, if the toxicity of ZnO is linked directly to Zn^2+^ ion, an inferior solubilization of particles could efficiently explain the toxicity values. The results after 48 h are reported in the Table 10 below.

The values shown in Table 10 give interesting information to explain the cytotoxicity mechanism and other chemical–physical characteristics of the ZnO–Ox complex. First of all, the solubilization is pH-dependent, according to Misra et al. [22]; as shown, the considerable increase in pH acts in favor of solubilization of ZnO particles. This could explain the behavior of Z potential shown in Figure 10; in fact, the negative values after isoelectric point could be explained through a solubilization of particles [12]. The second information given by this experiment regards the solubilization promoted by the environment; in fact, the DMEM medium shows an increase of zinc solubilization [22]. Only for non-nanometric ZnO–Ox (column 5, Table 10), the behavior was not confirmed; the reason could be explained by strong complexation of Oxisol with zinc that does not consent the presence of free Zn^2+^ in solution promoted by DMEM, mimics of the biological environment. The low biodisponibility of Zn^2+^, together with its reduced presence in functionalized samples, consent to understand better the low toxicity of the newly obtained complex in non-nanometric form. Differently, the nanometric ZnO–Ox shows higher solubilization (DMEM), but, at the same time, a very low toxicity with an inhibition of cellular grow around 4%; this means that other mechanisms, apart from solubilization, contribute to a decrease of toxicity, and it is likely possible that a release of ligand promoted by biological environment consent the antioxidant activity of Oxisol, then a decreasing of toxicity. Further investigations are in progress in our laboratory to assess this hypothesis.

Comparing the solubility data with Oxisol release reported in Table 6, the pH that gives the higher solubility corresponds to the lowest release of Oxisol (pH 3). Moreover, the maximum release of Oxisol was observed for pH 12, as expected. In fact, basic pH catalyzes the hydrolysis and, at the same time, the salification of Oxisol that promotes it solubilization in water. Contrary to what has been said for alkaline environment, an acid pH, despite it catalyzing the hydrolysis, does not allow the solubilization of Oxisol.

The solubility behavior shows a secondary peak around pH 9 (Table 9), despite that this pH value should not lead to a solubilization of ZnO [23].

### 2.8. Anti-Acne Activity

The cytotoxicity data obtained with ZnO led to hypothesize a possible use of adducts for acne contrast. In order to verify this, the inhibition of *Propionibacterium acnes* was evaluated by means of the minimum inhibitory concentration (MIC) and minimum bactericidal concentration (MBC) methods.

The adduct was compared to the Oxisol molecule as such. Only non-nanometric ZnO was tested, because of its larger use in the dermo-cosmetic field. The results are shown in Table 11 and Table 12.

This experiment confirmed the anti-acne activity of zinc, in particular ZnO. Furthermore, the efficiency against *C. acnes* was considerably increased by means of the functionalization of ZnO particles. The reason behind this increased activity is also under investigation; we may advance the hypothesis that it could be related to an increase of permeability of coated ZnO through bacteria wall by functionalization, as reported by Zhongbing and coworkers [24].

## 3. Materials and Methods

### 3.1. Materials

Zinc oxide (EverZinc, Liège, Belgium), Oxisol (Kalichem, Brescia, Italy), Acid Blue 9 (Farmalabor, Milan, Italy), Ethanol BioUltra, for molecular biology, ≥99.8% (absolute alcohol, without additive) (Sigma-Aldrich, Saint Louis, MO, USA), physiological solution 0.9% NaCl (S.A.L.F. Spa, Bergamo, Italy), UV-Vis spectrophotometer Jasco V-730 (Jasco, Mary’s Court, Easton, MD, USA), FT-IR spectrophotometer ATR FT-IR Jasco 4600, ATR PRO ONE (Jasco, Mary’s Court Easton), Centrifuge RE.MI XS R-8D (REMI, Mumbai, India), Turboemulsifier Silverson^®^ L5M-A (Silverson^®^, Evry, France), Viscosimeter Brookfield DV2T (Brookfield, Toronto, Canada), Densimeter Mettler Toledo DA-100M (Mettler Toledo, Columbus, OH, USA), Plaster 5 × 5 (Leukofix, Hamburg, Germany), Magnetic stirrer (Heidolph, Schwabach, Germany), SHIMADZU UV-2600 spectrophotometer provided of integrating sphere ISR 2600 60 mm, WW5 PMMA plates (Schonberg GmbH, Munich, Germany), Suntest CPSþ (Atlas, Linsengericht, Germany), LUMiSizer^®^ (L.U.M.GmbH, Berlin, Germany), Nicomp ZLS Z3000 (PSS, Port Richey, FL, USA). Netzsch 409/C (Gebrüder-Netzsch-Straße 1995100 Selb, Germany), Nicomp ZLS Z3000 (8203 Kristel Circle, Port Richey, FL, USA), Zeiss Sigma VP Field Emission SEM (Oberkochen, Germany), ICP–MS NexION 350D (Perkin Elmer, Waltham, MA, USA), Discover SP-D oven (CEM Corporation, Matthews, NC, USA). 

### 3.2. Zinc Oxide (ZnO) Functionalization: ZnO–Ox

This reaction was carried out on both nanometric and non-nanometric ZnO. In a 250 mL two-neck flask, 0.269 g of Oxisol was solubilized in ethanol with a bubble condenser, maintaining the temperature around 50 °C. After having wrapped the flask with tinfoil (to avoid photocatalytic reactions), the physical filter was added, and the mixture left under stirring for at least 1 h. The reaction was monitored via UV-Vis spectroscopy and characterized by IR.

### 3.3. Synthesis of Zn^2+^–Oxisol Complex

The synthesis was based on the method of Singh et al. [25]. In a 250 mL two-necked flask, 0.5 g of Oxisol was solubilized in water with 2 equivalents of NaOH. After complete solubilization, 3 equivalents of ZnCl_2_ was added (0.757 g). A pale brown precipitate was observed. After 24 h of reaction, we proceeded with centrifugation and exsiccation.

### 3.4. Evaluation of Stability of Oxisol–ZnO Particles

To start, 0.01 g of ZnO was placed in a 100 mL flask and brought up to volume with ethanol, ethanol/water (2:3) in a mixture with different pH (2.7, 6.1, 12), and water with pH 2.7, 6.1, and 12. The system was then stirred continuously and spectrophotometrically monitored to perform the leaching test at 30-min intervals for 4 h. At each time, 0.5 mL of solution was taken, centrifuged, filtered with a 0.45 micron filter, and diluted directly in the cuvette to 3 mL with the appropriate solvent. 

### 3.5. Sedimentation Rate

In order to check the stability of the dispersed system, the multi-sample analytical centrifuge LUMiSizer^®^ (L.U.M. GmbH, Berlin Germany) was used. The different behaviors of the single samples can be compared and analyzed in detail by detecting the transmission variation in any part of the sample or by tracing the movement of any phase limit [26]. Each sample was analyzed in triplicate, at a concentration of 10 mg/L. The analysis was carried out by placing the sample in a polycarbonate cuvette. The measurement parameters were set at a speed of 2000 rpm at 470 nm of laser wavelength and 600 profile numbers were detected every 10 s.

### 3.6. Zn Release from the Zinc-Based Materials (ZnO–Ox and Zn@Oxisol)

Stock solution of each Zn-based material was prepared and probe sonicated for 5 min. Aliquots of these suspensions were diluted in five different media (pH 3, 5, 7, 9, 12, and DMEM), obtaining samples with initial concentration of 10 mg L^−1^. These suspensions were stirred by a bench shaker at 400 rpm and an aliquot at different timing (0, 6, 24, and 48 h) was collected in PP vials and centrifuged at 18,407 RCF for 40 min following the procedure described by Wang et al. [27]. Then, the clear supernatants were carefully collected and acidified with 2% nitric acid and analyzed through ICP–MS. 

### 3.7. Digestion Analysis (ICP–MS)

The Zn content of each Zn base material was measured by means of ICP–MS (NexION 350D, Perkin Elmer), after a microwave acidic mineralization using a Discover SP-D oven (CEM Corporation). According to Badetti and co-workers [28], about 20 mg of dry samples was treated with a mixture of ultrapure H_2_O_2_ and HNO_3_ in a 1:2 ratio. The heating program used for the acid digestion was: T_MAX_ = 170 °C, ramp time = 5 min, hold time = 2 min, power = 300 W. Afterwards, the samples were cooled down for 30 min at room temperature, properly diluted, and analyzed by ICP–MS.

### 3.8. Photocatalysis

The photocatalytic activity was performed by monitoring the degradation following UV radiation of Acid Blue 9 mixed with pure metal oxide and coated metal oxide [17,18,19], both in conditions of light and darkness, in order to remove the contribution of dye adsorption and compared with the solution of Acid Blue 9 in the same conditions. Two identical solutions were prepared consisting of 10 mg of photocatalytic material in 100 mL of Acid Blue 9 0.77 mM in EtOH: One mixture was exposed to UV radiation (360–400 nm), the other was placed in conditions of darkness. At the end of this 1-h passage, the sample was left to rest for 3 h, subsequently centrifuged and filtered (0.45 micron), before proceeding with the UV-Vis analysis (628 nm).

### 3.9. Formulation

The study was carried out on different emulsions. The formulations containing Oxisol had a minimum percentage of 0.5% (to consent a booster effect), when either mixed or functionalized (ZnO–Ox). O/W emulsions were prepared using a turbo emulsifier; for this operation, the Turboemulsifier Silverson^®^ L5M-A (Silverson^®^, Evry, France) was used.

Each study product used the same base formulation in order to minimize errors. The formulations compositions are as follows: 

Phase A: Steareth-21, Steareth-2, glyceryl monostearate, cetearyl alcohol, benzyl alcohol–dehydroacetic acid, coco-caprylate, dihydroxyphenylbenzimidazole carboxylic acid (Oxisol) (when required);

Phase B: Nanometric ZnO or non-nanometric ZnO, or nanometric ZnO–Ox or non-nanometric ZnO–Ox;

Phase C: Panthenol, NaOH, xanthan Gum, aqua.

The base formulation was prepared by mixing the ingredients of phase A, except for the preservation system (benzyl alcohol–dehydroacetic acid), Oxisol (when required), and coco-caprylate, then heating until melting. Phase C was prepared separately with the addition of 90% of water, brought to 80–90 °C, and mixing until complete solvation of the xanthan gum. When 80 °C was reached for each phase, phase C was added to A, stirring continuously. The base thus obtained was maintained at 70 °C before proceeding with functionalization, which was carried out differently depending on the formulation:

(1) Formulations without Oxisol: To the coco-caprylate was added the powder of phase B, then proceeded to mix with force until obtaining a homogeneous dispersion. Under continuous mixing, the obtained dispersion was gradually added to the base formulation, until a homogeneous emulsion was obtained. Finally, the remaining water was added until homogenization.

(2) Formulation containing Oxisol alone or as ZnO–Ox: The powder of phase B was mixed with coco-caprylate, as reported in point (1). Oxisol was solubilized separately in water by quenching with NaOH. The powder dispersion was then added to the base formulation, under continuous mixing, and finally the Oxisol solution was also added.

The pH and viscosity of each preparation were evaluated. The pH of the emulsions was adjusted within a range of 5.5–6.5. If required, the pH was corrected with the addition of citric acid (Table 13).

### 3.10. Characterization

#### 3.10.1. FT-IR Analysis

The FT-IR spectra were collected in the 4000–650 cm^−1^ range, with a resolution of 5 cm^−1^ at room temperature by using a Jasco FT/IR-4600 spectrometer provided with single ATR accessory (Jasco ATR PRO ONE). The number of accumulated scans for each recorded spectrum was automatic, with an average exposure around 1.5 min.

#### 3.10.2. Thermo-Gravimetric Analysis (TGA) and Differential Scanning Calorimetry (DSC)

TGA and DSC were performed simultaneously with Netzsch 409/C. The heating program included an increase from 30 to 1000 °C, with an increase of 5 °C min^−1^. About 15 mg of each sample was placed in a platinum/rhodium crucible using alumina for internal calibration. Measurements were performed in air/N_2_ mixture (40/80 mL/min).

#### 3.10.3. ζ Potential (DLS–ELS)

The hydrodynamic diameter was measured by means of a multi-angle Nicomp ZLS Z3000. The dried powders were dispersed again in ultrapure water (10 mg/L) by probe sonicating in ice bath for 10 min (80% pulsed mode, 200 W). All measurements were taken after a 5-min pre-balance. The dispersion light was collected with an optical fiber set at a dispersion angle of 90° (W = 25 mW and λ = 639 nm) for at least 6 min at room temperature.

The potential zeta characterization by electrophoretic light scattering (ELS) was achieved by Nicomp ZLS Z3000 (PSS, Port Richey, FL, USA). The zeta potential values (Z-pot) of each sample were determined in a range of pH 3–11, using NaCl as electrolyte (10 mM), as reported by Brunelli et al. [29].

#### 3.10.4. SEM

The materials were suspended in EtOH (0.1–0.5 mg/mL final concentration) and probe sonicated. About 3 μL of the suspension was deposited on a silicon wafer substrate and dried at 60 °C for 12 h. Images were collected in high vacuum with a Zeiss Sigma VP Field Emission SEM, using an in-lens detector at 5.0 keV beam energy. The elemental composition of a sample was determined using characteristic X-ray spectrum of the specimen being examined. The EDS analysis was performed in a ‘‘spot mode’’ in which the beam (set at 10 keV) was localized on a single area manually chosen within the field of view. The location was represented on the provided SEM images by a ‘‘+’’. Data was elaborated by means of Bruker Esprit 1.9.

### 3.11. Cytotoxicity

ZnO cytotoxicity was measured by neutral red dosage (NRU) performed in DMEM (Dulbecco’s Modified Eagle Medium) supplemented with 10% fetal calf serum (FCS), penicillin (100 U/mL), streptomycin (100 μg/mL), and glutamine (2 mM). The cells were seeded in triplicate in 96-well plates with a density of 7–103 cells/well and treated for 48 h with increasing concentrations of the different compounds (1, 10, and 100 μg/mL). Untreated cells were used as a negative control. The cells, after treatment, were washed and added with 250 μL of a solution of 25 μg/mL of NRU. After 2 h, the cells were washed again and 150 μL of NRU-desorb solution (49% water, 50% ethanol 95%, and 1% glacial acetic acid) were added. After another 40 min, the absorbance of the solution at 540 nm was measured by spectrophotometer and converted to % of growth inhibition.

### 3.12. Anti-Acne Activity: Determination of MIC (Minimum Inhibitory Concentration) and MBC (Minimum Bactericidal Concentration)

Tryptic soy broth (TSBroth) with 5% sheep blood (defibrinated) was used as culture media for the preparation of the bladder suspension and to perform suspension tests. Tryptic soy agar (TSA) with 5% sheep blood (defibrinated) was used to perform surface tests (diffusion agar). The Anaerocult^®^ system was used for 5-da-long anaerobiosis incubation at 37 °C.

The minimum inhibitory concentration of the organism growth, defined as MIC, and the minimum bacterial concentration, defined as MBC, were evaluated in microtiter plates containing a microbial inoculum at a concentration of 106, corresponding to the turbidity of 0.5 Mac Farland, obtained from standard overnight bacteria cultures in TSBroth with 5% sheep blood (defibrinated).

The antimicrobial potency was evaluated by the inhibition of microbial growth (*Cutibacterium acnes*) on the agarized soil. Acne causing test microorganism *Cutibacterium acnes* (NCTC 737) was purchased from Public Health England (PHE) Culture collections: *Cutibacterium acnes*, ATCC 6919. 

Nitrocellulose discs (DN) with a diameter of 1.0 cm were placed in contact with each test product for 30 min, dried for 30 s and then placed, sufficiently spaced apart, on plates prepared with agar culture medium previously sown with the microbial suspension test of *Cutibacterium acnes*.

Finally, the diameter of the inhibition halo of the pathogenic bacteria *Cutibacterium acnes* was measured, after anaerobic incubation for 5 days at 37 °C.

The suspension method is a quantitative in vitro method for assessing the sensitivity of the microbial strain to a product with antimicrobial activity. The evaluation was carried out via turbidimetry on the microbial suspension. Subsequently, the microbial survival was evaluated via total inclusion microbial count. The product under examination was divided into wells, then contaminated with the known titration of *Cutibacterium acnes* microbial suspension. The survival of *Cutibacterium acnes* was evaluated by TSA with 5% sheep blood (defibrinated) plate inclusion method, via incubation, under anaerobic conditions.

In the microtiter plate, each well was prepared with the BROTH enrichment medium with 5% sheep blood (defibrinated), usually 999 μL of soil per well. Then, 1 μL of the antimicrobial activity sample was add into the well. The tests were repeated in triplicate. Once the microtiter plates were prepared, the inoculum with the *Cutibacterium acnes* was added. The inoculum was prepared using a standard culture developed at +37 °C for 96 h anaerobiosis. The microbial concentration of the inoculum was 1 million per milliliter (corresponding to opacity of 0.5 McFarland, or 1.5 × 10^6^ bacteria).

The seeded microtiter plates were incubated in anaerobiosis for 5 days at +37 °C, in order to evaluate the turbidity of the well as an index of bacterial colony development. Results are defined considering the turbidity of the well, which was used to calculate the MBC value or minimum bactericidal concentration. Each well, corresponding to the dilution test, was subsequently analyzed by the inclusion method in agar and incubated under anaerobiosis conditions for 5 days at +37 °C.

Each test was performed in triplicate and the growth of *Cutibacterium acnes* bacteria was checked every day of anaerobic incubation until the end of the incubation time.

### 3.13. Oxisol Release from the Emulsions

Approximately 0.30 g of emulsion weighed exactly was placed in a 50 mL flask, brought to volume with physiological solution and left under stirring for 4 h at 37 °C. Then, 10 mL of solution was then quantitatively transferred into a test tube, centrifuged for 10 min at 6000 rpm, and filtered. Lastly, 0.5 mL of this solution was diluted to 3 mL with distilled water directly in the cuvette and analyzed by spectrophotometer.

### 3.14. Photochemiluminescence (PCL)

The PCL assay measures the antioxidant activity of a sample against superoxide anion radicals generated from luminol, a photo-sensitizer, when exposed to UV light (Double Bore^®^ phosphor lamp, output 351 nm, 3 mWatt/cm^2^), and is based on Popov and Lewin’s method [30,31]. Measurements were carried out with a Photochem^®^ apparatus (Analytik Jena, Leipzig, Germany). The antioxidant activity was measured using Antioxidant Capacity of Liposoluble (ACL) substance kits provided by the manufacturer [32]. The Luminol reagent and Trolox work solution were freshly prepared according to the ACL protocol. The kinetic light emission curve was monitored for 180 s and expressed as micromoles of Trolox per gram of dry matter. The areas under the curves were calculated using the PCLsoft control and analysis software. A calibration curve was constructed with scalar Trolox concentrations: The different reduction in signal amplitude was used as a parameter for quantification and correlated to the reduction of the integral of PCL intensities caused by the different concentrations of Trolox. The concentration of the individual samples must be such that the luminescence generated during the 180-s sampling interval is within the limits of the standard curve. The results are expressed in micromoles of Trolox^®^, which provide an antioxidant capacity equivalent to 1 g of the sample under examination.

### 3.15. In Vitro Evaluation of Filtering Parameters

The method used in this work was recently proposed by us, adapting the ISO 24443:2012 standard for the determination of UVA protection in vitro to UVB [33].

In vitro SPF spectrophotometric evaluation was performed measuring transmittance spectrum by a SHIMADZU UV-2600 spectrophotometer provided of integrating sphere ISR 2600 60 mm and coupled with an SPF determination software (SPF calculator software version 2.1, Shimadzu, Milan, Italy) to obtain the values of SPF, UVA/UVB, UVAPF, and λ critical.

The in vitro approach used consisted of applying a thin film of product on an artificial substrate. The physical characteristics of this substrate must be as similar as possible to human skin. For this reason, the substrate used for this study was PMMA plates (WW5 PMMA plates have been purchased from Schonberg GmbH, Munich, Germany), a substrate easily handled and that can be supplied with a 5 μm reproducible roughness, with an area of 25 cm^2^. Via spectrophotometric measures, the amount of UV radiation passing through the film can be evaluated. Photostability studies were carried out with a solar simulator device (Suntest CPS; Atlas, Linsengericht, Germany) equipped with a Xenon lamp, an optical filter to cut off wavelengths shorter than 290 nm, and an IR-block filter to avoid thermal effects, as prescribed by the ISO 24443:2012 procedure.

### 3.16. Statistical Evaluations

The analysis comparison was carried out considering *p*-values that represent the probability of finding the observed, or more extreme, results when the null hypothesis (H_0_) of a study question is true. The choice of significance level at which H0 is reject is arbitrary. Conventionally, the 5% (less than 1 in 20 chance of being wrong), 1%, and 0.1% (*p* < 0.05, 0.01, and 0.001) levels were used. Most authors refer to statistically significant *as p* < 0.05 and statistically highly significant *as p* < 0.001 (less than 1 in 1000 chance of being wrong). 

## 4. Conclusions

This study aimed to investigate the possible formation of adducts of dihydroxyphenyl benzimidazole carboxylic acid (Oxisol) to ZnO in order to compare some of our interesting results previously obtained by physical mixtures of the two. To this end, a synthetic approach was developed to obtain ZnO and Oxisol linked by a covalent bond. Theoretically, the two possible types of product obtainable with our approach are coating and complexation; however, simple coating is not sufficient to explain the obtained results. In fact, ATR–FT-IR, SEM, and SEM–EDX analysis showed a complexation of ZnO instead of coating, which means a covalent bond with different properties than a simple coated particle. The addition of Oxisol was favored in the case of ZnO non-nanometric form, instead of nanometric form; this can be explained by a large size population of the sample (Table 4) from 10 to 1600 nm. However, although what was obtained is reasonably a mixture of complexed and coated material, good efficiency was achieved in improving the UV protection: In fact, the SPF values increased more than 100% for nanometric and around 60% for non-nanometric form.

On the other hand, the involvement of the catechol hydroxyl groups of Oxisol in the binding to ZnO particles almost suppressed its antioxidant activity. Nevertheless, photocatalytic activity of ZnO showed an important inhibition, which can be attributed to the complexation, besides the activity of the ligand on band gap. An expected behavior was shown by Z potential, with a strong decrease at low pH values, because of the solubility of ZnO particles in this pH region. However, sedimentation rate showed a good result for the product obtained from non-nanometric form and worst for the nanometric one. We hypothesized that needle-shape crystal observed in the case of non-nanometric ZnO–Ox could improve dispersion stabilization.

The cytotoxicity study offered a valid interpretation of the behavior of inorganic sunscreens in contact with cells. The most interesting results were observed in the experiment with sample concentration at 10 μg/mL: It is very interesting to note that the non-functionalized zinc oxide showed a toxicity that reached over 50% of the inhibition of cell growth; instead, the complexation reduced inhibition by 10 times to only around 5%. We believe that this cytotoxicity is due to the presence of Zn^2+^ ion in solution, as proved by the solubility test. The reduced cytotoxicity of ZnO but not the complete annulment suggests an application as an anti-acne agent. The results are very promising, with a significant increase of activity against acne as compared with pure ZnO. 

In light of these unprecedented results, we can conclude that the new ZnO adducts described above have several benefits over the simple ZnO behavior: The stabilization in formulation and the booster activity allowed to reduce the quantity of inorganic filter in UV sunscreens; moreover, the reduced photocatalytic activity and cytotoxicity led to an improved toxic profile of ZnO. Finally, the activity against *C. acne* showed a consistent anti-acne activity, also when compared with pure ZnO. Thus, our investigation leads not only to a new class of mixed inorganic filters, but also to obtain very important information regarding the cytotoxicity of ZnO and the mechanism at the base of this action. In particular, Oxisol showed a high activity of free Zn^2+^ coordination, strongly decreasing the cytotoxicity of zinc oxide.

## 5. Patents

The content of the present work has been filed by the University of Ferrara, patent appl. n.102019000014076, 8 August 2019.

## Figures and Tables

**Table 1:** TGA values obtained for ZnO. For each method, analysis was performed three times.

	Weight Loss Referred to Organic Fraction (%)
Nanometric ZnO–Ox	21.08 ± 0.8
Non-nanometric ZnO–Ox	42.26 ± 1.1
Zn complex	78.67 ± 0.1

**Table 2:** Organic fraction measured by ICP. The degree was obtained by the difference between Zn^2+^ ion percentage in pure particles and functionalized particles.

	Organic Fraction (%)
Nanometric ZnO–Ox	26.44 ± 1.3
Non-nanometric ZnO–Ox	38.16 ± 2.3
Zn complex	77.69 ± 1.4

**Table 3:** The first and second columns show the particle size values detected via DLS and CSA, ± Standard Deviation (SD). The last column shows the sedimentation rate. Because of the great inhomogeneities of results given by non-nanometric ZnO, we carried out a further analysis three times; this consented us to find three different particle populations, from 10 to 1600 nm. The analysis timing was: t_0_, t_1_ (0.5 h), t_3_ (1.0 h).

	DLS (nm) ± SD	CSA (nm) ± SD	Sedimentation Rate (μm/s) ± SD
Non nanometric ZnO	1612 ± 290 411.4 ± 59 10.7 ± 1.5	247.27 ± 15.2	94.22 ± 0.5
Non-nanometric ZnO–Ox	162 ± 36	135.37 ± 1.9	35.90 ± 1.5
Nanometric ZnO	167 ± 34	162.63 ± 1.2	33.91 ± 0.3
Nanometric ZnO–Ox	46 ± 39	164.63 ± 3.2	38.67 ± 0.8

**Table 4:** UV filtering parameters pre- and post-irradiation.

**A. Pre-Irradiation**	**SPF**	**UVA-PF**	**UVA/UVB**	**ʎ** **c**
Emulsion base	0.99 ± 0.04	0.98 ± 0.04	0.99 ± 0.04	290 ± 1.00
Emulsion with Oxisol	1.31 ± 0.12	1.17 ± 0.11	1.00 ± 0.1	370 ± 1.00
Emulsion with non-nanometric ZnO	1.62 ± 0.15	1.60 ± 0.15	1.00 ± 0.12	377 ± 1.00
Emulsion mixture (Non nanometric ZnO+Oxisol)	2.08 ± 0.15	2.10 ± 0.17	1.03 ± 0.15	381 ± 1.00
Emulsion with non-nanometric ZnO–Ox	3.32 ± 0.28	2.50 ± 0.24	0.93 ± 0.15	379 ± 1.00
Emulsion with nanometric ZnO	1.58 ± 0.15	1.55 ± 0.15	1.00 ± 0.12	374 ± 1.00
Emulsion with nanometric ZnO–Ox	2.01 ± 0.18	2.08 ± 0.19	1.04 ± 0.13	381 ± 1.00
Emulsion mixture (Nanometric ZnO+Oxisol)	4.17 ± 0.34	2.65 ± 0.27	0.86 ± 0.15	375 ± 1.00
**B. Post-irradiation**	**SPF**	**UVA-PF**	**UVA/UVB**	**ʎ** **c**
Emulsion base	0.98 ± 0.04	0.97 ± 0.05	0.99 ± 0.04	290 ± 1.00
Emulsion with Oxisol	1.21 ± 0.13	1.10 ± 0.15	0.91 ± 0.1	369 ± 1.00
Emulsion with non-nanometric ZnO	1.51 ± 0.16	1.50 ± 0.17	1.00 ± 0.12	376 ± 1.00
Emulsion mixture (Non nanometric ZnO+Oxisol)	1.91 ± 0.15	1.95 ± 0.17	1.02 ± 0.15	383 ± 1.00
Emulsion with non-nanometric ZnO–Ox	3.02 ± 0.31	2.33 ± 0.35	0.77 ± 0.15	375 ± 1.00
Emulsion with nanometric ZnO	1.47 ± 0.13	1.46 ± 0.15	0.99 ± 0.12	375 ± 1.00
Emulsion with nanometric ZnO–Ox	1.87 ± 0.20	1.93 ± 0.21	1.03 ± 0.13	382 ± 1.00
Emulsion mixture (Nanometric ZnO+Oxisol)	3.88 ± 0.32	2.49 ± 0.33	0.64 ± 0.15	372 ± 1.00

**Table 5:** PCL values for ZnO emulsions.

Formulation	μmoli TE/Gram
Emulsion with Oxisol 0.5%	41.96 ± 2.07
Emulsion mixture (Non nanometric ZnO+Oxisol)	1.31 ± 0.06
Emulsion mixture (Naometric ZnO+Oxisol)	1.81 ± 0.07
Emulsion with non-nanometric ZnO–Ox	1.98 ± 0.05
Emulsion with nanometric ZnO–Ox	1.65 ± 0.04

**Table 6:** Oxisol release from particles in different solvents and pH value for ZnO.

Substrate	Solvent	pH	Time (h)	Oxisol Desorption (%)
		12.0		<1.0
	CH_3_CH_2_OH/H_2_O	6.1		<1.0
		2.7		<1.0
Nanometric	CH_3_CH_2_OH	-	4.0 ± 0.5	<1.0
ZnO		12.0		23.49 ± 1.55
	H_2_O	6.1		9.01 ± 1.80
		2.7		<1.0
		12.0		<1.0
	CH_3_CH_2_OH/H_2_O	6.1		<1.0
Non-		2.7		<1.0
nanometric	CH_3_CH_2_OH	-	4.0 ± 0.5	<1.0
ZnO		12.0		20.76 ± 1.21
	H_2_O	6.1		6.72 ± 1.34
		2.7		<1.0

**Table 7:** Oxisol percentage released. The values are referred to Oxisol (%) functionalized on ZnO (ZnO–Ox).

Substrate	Issue of Release of the Emulsion Adduct	Time (h)	Oxisol Released in the Emulsion Test (%)
Nanometric ZnO–Ox	H_2_O 0.90% NaCl	4.0 ± 0.5	8.73 ± 0.34
Non nanometric ZnO–Ox	H_2_O 0.90% NaCl	4.0 ± 0.5	7.20 ± 0.31

**Table 8:** Photocatalytic activity for pure dye, functionalized ZnO–Ox, and untreated ZnO. The values are reported as micromolar (μM) after treatment.

	**Concentration (μM)**	
Only Acid Blue 9 solution (dark)	109.99 ± 23.59	
Only Acid Blue 9 solution (UV)	86.50 ± 18.86	
	**Dye Concentration (μM) (nanometric ZnO)**	**Dye Concentration (μM) (non-nanometric ZnO)**
ZnO–Ox (dark)	93.68 ± 20.35	73.07 ± 16.13
ZnO–Ox (UV)	90.91 ± 19.73	74.77 ± 16.50
ZnO (dark)	91.22 ± 19.86	86.20 ± 18.86
ZnO (UV)	2.50 ± 2.08	2.11 ± 1.95

**Table 9:** Cytotoxicity values (cell growth inhibition) obtained evaluating the different concentration of products (from 10 to 100 μg/mL).

Sample	Concentration (μg/mL)	% Inhibition ± Standard Deviation
Control	0	0.00 ± 0.00
Nanometric ZnO	1	0.15 ± 6.56
	10	61.11 ± 12.30
	100	87.89 ± 0.81
Nanometric ZnO–Ox	1	−9.33 ± 3.80
	10	4.20 ± 4.56
	100	88.02 ± 0.80
Non-nanometric ZnO	1	−3.67 ± 5.53
	10	53.25 ± 16.93
	100	87.86 ± 0.85
Non-nanometric ZnO–Ox	1	−11.23 ± 6.95
	10	6.58 ± 3.23
	100	87.82 ± 0.96

**Table 10:** Zn^2+^ present in solution after 48 h under different conditions. Values are reported in percent (%). The value corresponding to 100% was attributed by the samples subjected to digestion.

	Zn^2+^ (%)			
	Nanometric ZnO	Non Nanometric ZnO	Nanometric ZnO–Ox	Non-Nanometric ZnO–Ox
	85.05 ± 6.0	77.67 ± 6.2	58.61 ± 2.9	39.51 ± 2.4
Analyzed condition	Zn^2+^ in Solution (%) **			
pH 3	94 ± 6	100 ± 3	102 ± 2	103 ± 2
pH 5	14 ± 1	15 ± 1	36 ± 2	60 ± 3
pH 7	18 ± 1	12 ± 1	34 ± 1	57 ± 2
pH 9	27 ± 1	31 ± 1	42 ± 1	52 ± 2
pH 12	0 ± 0	1 ± 0	7 ± 1	11 ± 1
DMEM * (pH 7.4)	75 ± 4	81 ± 3	81 ± 3	55 ± 1

* DMEM is supplemented with 10% fetal calf serum (FCS), penicillin (100 U/mL), streptomycin (100 μg/mL), and glutamine (2 mM), Section 4. ** Zn^2+^ percentage normalized to effective sample concentration.

**Table 11:** Anti-acne activity obtained via minimum inhibitory concentration (MIC). * = not efficacious (N); + = low sensitivity (L); ++ = intermediately susceptible (M); +++ = sensitive (S). Each value is statistically significant.

	Microorganism *Cutibacterium Acnes* (1 μg/mL)	Diameter	Inhibition (Halo Interpretation *)	Antimicrobial Activity Result *
ZnO–Ox	6.00 × 10^6^	4.0	+++	S
Oxisol	6.00 × 10^6^	1.9	+	L
ZnO	6.00 × 10^6^	2.4	++	M

**Table 12:** Anti-acne activity obtained via minimum bactericidal concentration (MBC). * 0% = not efficacy (N); <90% = low sensitivity; 98.9–90.0% = intermediately susceptible; 99.0–99.999% = sensitive. Each value is statistically significant.

	Microorganism *Cutibacterium Acnes* (1 μg/mL)	Result after Contact Time T1	Reduction	Antimicrobial Activity Result *
ZnO–Ox	6.00 × 10^6^	6.40 × 10^4^	98.97%	M
Oxisol^®^	6.00 × 10^6^	2.70 × 10^6^	55.00%	L
ZnO	6.00 × 10^6^	5.80 × 10^5^	90.33%	M

**Table 13:** Chemical–physical parameters for each formulation. Viscosity parameters are: Spindle 25, 10 rpm, detection 30”.

Formulation	pH	Viscosity (ŋ) cP
Only ZnO	6.53	11,230
ZnO + Oxisol (mixture)	6.67	29,520
Functionalized ZnO (ZnO–Ox)	6.50	39,650
Only ZnO nanosized	6.68	14,160
ZnO nanosized + Oxisol (mixture)	6.50	16,420
Functionalized ZnO nanosized (nanometric ZnO–Ox)	6.00	17,860
